# Mental health law across the UK^†^

**DOI:** 10.1192/pb.bp.117.056622

**Published:** 2017-12

**Authors:** Tony Zigmond

**Affiliations:** 1 Retired consultant psychiatrist

## Abstract

The criteria governing medical treatment without consent in the three legal jurisdictions of the UK – England and Wales, Scotland and Northern Ireland – is discussed.

‘A doctor is not that sinister figure which in former times he was represented to be, anxious simply to confine a man in a dungeon for life. He is treating mental disorder in exactly the same way as he treats any other disease.’

Lord Russell 1928^1^

Nearly 20 years ago I wrote an editorial in this journal that there should be a medical incapacity act to ‘provide for the medical treatment, both mental and physical, of those who lack capacity from whatever cause. It would establish a statutory framework offering the same protections to all patients who are unable to consent to medical intervention, from both physical and psychiatric conditions, and permit investigation and treatment of both the physical and mental illnesses of such patients’.^2^ Szmuckler & Holloway have not just argued for a single piece of legislation for all nonconsensual care and treatment saying that a mental health act is harmful,^3^ they have written a draft act under the heading of ‘Fusion Law’.^4^ We may soon know whether we were right.

There are three distinct legal jurisdictions within the UK: England and Wales, Northern Ireland and Scotland. Until recently all had a mental health act to regulate the care and treatment of people with a mental illness while relying on common law to do the same for the physically ill. The powers given to doctors and other healthcare professionals by the two regimes was very different.

Within a year or two either side of the turn of this century, all the jurisdictions started to review their relevant legislation, not only their mental health law but also the necessity for replacing common law with statutory provision for the non-consensual treatment of physical illness, not least to ensure compliance with the European Convention on Human Rights as required by the Human Rights Act 1998. Scotland passed its Adults with Incapacity (Scotland) Act 2000, England and Wales its Mental Capacity Act 2005, Northern Ireland its Mental Capacity Act (Northern Ireland) 2016. For their mental health acts, each started with a review: Richardson for England and Wales published in 1999,^5^ Millan for Scotland in 2001^6^ and Bamford for Northern Ireland in 2007.^7^ Each considered whether or not their mental health act should include a ‘capacity’ criterion for medical treatment without consent.

The courts have been consistent about the role of capacity when adults make a decision about medical treatment. Lord Reid in 1972 said:

‘There is no doubt that a person of full age and capacity cannot be ordered to undergo a blood test against his will. … the real reason is that English law goes to great lengths to protect a person of full age and capacity from interference with his personal liberty. We have too often seen freedom disappear in other countries not only by coups d'etat but by gradual erosion: and often it is the first step that counts. So it would be unwise to make even minor concessions’.^8^

Lord Donaldson, then Master of the Rolls, in 1992 said:

‘Prima facie every adult has the right and capacity to decide whether or not he will accept medical treatment, even if a refusal may risk permanent injury to his health or even lead to premature death’.^9^

Ten years later Dame Butler-Sloss said the same:

‘A competent patient has an absolute right to refuse to consent to medical treatment for any reason, rational or irrational, or for no reason at all, even when that decision may lead to his or her death’.^10^

They all emphasised the importance of autonomy. Although common law has now been replaced by statute law, the provisions of capacity legislation fully reflect these sentiments. I note that all the Judges were, of course, incorrect. A competent person with a mental illness may have no right to refuse treatment.

To return to the reviews. The first principle of Recommendation 3.3 in the Millan report is ‘Non discrimination – People with mental disorder should whenever possible retain the same rights and entitlements as those with other health needs’ (p. 23).^6^ So, should capacity be a criterion as it is for those with other health needs? The report sets outs the arguments against the capacity criterion including that there were difficulties assessing capacity, particularly in patients with mood disorders, obsessive–compulsive disorders and eating disorders; ‘Such patients might retain legal capacity but be at such risk as to justify intervention’ (p.55).^6^ The committee was also told by some psychiatrists that

‘incapacity was a concept which they would find difficult to measure and apply. The British Medical Association (BMA) suggested that a capacity test would make it harder for GPs and doctors in, for example, Accident and Emergency Departments to come to a decision, and might lead to a reluctance to use the Act. The United Kingdom Central Council for Nursing, Midwifery and Health Visiting (UKCC), while in favour of making the justification for non-consensual interventions more explicit, suggested that professionals were not equipped to apply sophisticated tests of capacity fairly’ (p.55).^6^

These comments about difficulties assessing capacity are noteworthy because no individual or organisation said they could not operate or abide by the provisions of that country's incapacity act which, of course, requires assessment of capacity.

The Millan committee advised

‘that it should not be possible for a compulsory intervention to be made under mental health law unless there is evidence that the person's judgement is significantly impaired, as a result of mental disorder, so as to justify the intervention. This expresses a broadly similar concept to incapacity, but is felt to be a less legalistic formulation, and one which may be easier to apply in practice’ (p. 57).^6^

Significantly impaired decision-making as a result of the mental disorder (SIDMA) is a criterion for compulsion in the Mental Health (Care and Treatment) (Scotland) Act 2005. Millan had asked the question as to whether a mental health act was necessary and decided it was. Richardson also considered the question of capacity. Similar to Millan, ‘The principles governing mental health care should be the same as those which govern physical health’ (p. 21)^5^ wherever possible. Although deciding that incapacity should not be a criterion because ‘Mental disorder unlike most physical health problems may occasionally have wider consequences for the individual's family and carer, and very occasionally for unconnected members of the public’ (p. 19),^5^ the report suggested a form of words which would require the assessment of, and taking into account, the patient's capacity. Patients with capacity could only be detained if they presented a higher degree of risk compared with patients who lacked capacity. The government rejected this proposal. That a distinct mental health act was required was not questioned. There is no ‘capacity’ or ‘SIDMA’ criterion, nor a distinction between patients who retain capacity and those who do not, in the Mental Health Act 1983 (amended in 2007). To have such a criterion would have meant, according to Lord Hunt during the debate on the 2007 Bill, ‘abandoning one of the most fundamental objectives of the Act, namely that compulsory intervention should be based on need and risk’.^11^ To spell it out, in England and Wales a person can be forced to accept treatment for their mental illness if there is a health risk to themselves or others despite retaining full decision-making capacity. The equivalent law for physical illness, the Mental Capacity Act 2005, only applies if the person cannot consent because they lack capacity to do so. Patients with capacity who refuse consent cannot be forced to accept treatment no matter what the risks.

Northern Ireland has taken rather longer to determine their way forward. The outcome of the Bamford review and the provisions of their new act are detailed by Lynch *et al.*^12^There is to be no mental health act. The Mental Capacity Act (Northern Ireland) 2016 gives exactly the same legal framework for the non-consensual medical treatment of all citizens no matter what their illness.

Parity of esteem, a flagship policy of the Royal College of Psychiatrists, is best described, according to the College, as: ‘Valuing mental health equally with physical health’ (p. 3).^13^ The College has described a range of equalities which need to be achieved although, perhaps surprisingly, there is no mention of equality under the law. Northern Ireland now has equality under the law. The day after the judgment in the case of Ms B,^10^ *The Independent* newspaper wrote ‘Never again may a clinician administer treatment against the will of a mentally competent patient’.^14^ In Northern Ireland that will soon be true.

Earl Howe, in his closing statement during the passage of the Mental Health Bill 2007 (England and Wales), quoted the Millan committee, ‘It should not be the function of mental health law to impose treatment on those who are clearly able to make decisions for themselves’ and then continued, ‘As it is we are, in a real sense, back in the world of Enoch Powell and 1959. Patient empowerment and respect for the wishes of the patient are acknowledged features of good clinical practice in all other areas of healthcare – but not, it seems, in mental health’.^15^ The Mental Health (Northern Ireland) Act 2016 gives psychiatrists the same legal powers as all other doctors, and psychiatric patients the same autonomy and respect as all other patients. Where Northern Ireland has led surely other UK jurisdictions, and countries across the world, will follow.

## References

[1] Jones K Mental Health and Social Policy, 1845–1959. Routledge, reprinted 1998.

[2] Zigmond AS Medical incapacity act. Psychiatr Bull 1998; 22: 657–8.

[3] Szmukler G Holloway F Mental health legislation is now a harmful anachronism. Psychiatr Bull 1998; 22: 662–5.

[4] Szmuckler G Daw R Dawson J A model law fusing incapacity and mental health legislation. J Ment Health Law 2010; doi: http://dx.doi.org/10.19164/ijmhcl.v0i20.232 Available at: http://www.northumbriajournals.co.uk/index.php/IJMHMCL/article/view/232

[5] Richardson G Report of the Expert Committee: Review of the Mental Health Act 1983. Department of Health, 1999.

[6] Millan B New Directions: Report on the Review of the Mental Health (Scotland) Act 1984. Scottish Executive, 2001.

[7] Department of Health Northern Ireland The Bamford Review of Mental Health and Learning Disability (Northern Ireland): A Comprehensive Legislative Framework. Department of Health Northern Ireland, 2007.

[8] *S.v McC., W.v W*. [1972] A.C. 24, 43.

[9] *Re T (Adult: Refusal of Medical Treatment)* [1992] 4 All ER 649. 11648226

[10] *Re B (Adult: Refusal of Medical Treatment)* [2002] 2 All ER 449. 10.1136/jme.28.4.232 PMC1733608 12161574

[11] Hunt Lord Mental Health Bill. Hansard, 2 7 2007, Column 823.

[12] Lynch G Campbell P Taggart C Mental Capacity Act (Northern Ireland) 2016. BJPsych Bulletin 2017; doi: 10.1192/pb.bp.117.056945 (ePub ahead of print). 10.1192/pb.bp.117.056945 PMC5709686 29234514

[13] Royal College of Psychiatrists *Whole-person Care: From Rhetoric to Parity. Achieving Parity between Mental and Physical Health* (Occasional Paper OP88). Royal College of Psychiatrists, 2013.

[14] Boggan S Miss B: She smiled after her historic victory. But there was no joy, for now she is set to die. The Independent 2002; 23 3 (http://www.independent.co.uk/news/uk/crime/miss-b-she-smiled-after-her-historic-victory-but-there-was-no-joy-for-now-she-is-set-to-die-9227211.html).

[15] Howe Earl Mental Health Bill. Hansard, 2 7 2007, Column 825.

